# Extracellular ATP is a danger signal activating P2X7 receptor in a LPS mediated inflammation (ARDS/ALI)

**DOI:** 10.18632/oncotarget.25761

**Published:** 2018-07-17

**Authors:** Sanja Cicko, Thomas Christian Köhler, Cemil Korcan Ayata, Tobias Müller, Nicolas Ehrat, Anja Meyer, Madelon Hossfeld, Andreas Zech, Francesco Di Virgilio, Marco Idzko

**Affiliations:** ^1^ University Hospital Freiburg, Department of Pneumology, Freiburg, Germany; ^2^ Division of Pneumology, University Hospital RWTH Aachen, Aachen, Germany; ^3^ Department of Experimental and Diagnostic Medicine, University of Ferrara, Ferrara, Italy

**Keywords:** acute respiratory distress syndrome, ATP, KN62, P2X7, celltype specific P2X7KO

## Abstract

Acute respiratory distress syndrome (ARDS) is a life-threating lung condition resulting from a direct and indirect injury to the lungs [1, 2]. Pathophysiologically it is characterized by an acute alveolar damage, an increased permeability of the microvascular-barrier, leading to protein-rich pulmonary edema and subsequent impairment of arterial oxygenation and respiratory failure [1]. This study examined the role of extracellular ATP in recruiting inflammatory cells to the lung after induction of acute lung injury with lipopolysaccharide (LPS). However, the precise mechanism is poorly understood. Our objective was to investigate the functional role of the P2X7 receptor in the pathogenesis of acute respiratory distress syndrome (ARDS/ acute lung injury (ALI)) *in vitro* and *in vivo*. We show that intratracheally applied LPS causes an acute accumulation of ATP in the BALF (bronchoalveolar lavage) and lungs of mice. Prophylactic and therapeutic inhibition of P2X7R signalling by a specific antagonist and knock-out experiments was able to ameliorate the inflammatory response demonstrated by reduced ATP-levels, number of neutrophils and concentration of pro-inflammatory cytokine levels in the BALF. Experiments with chimeric mice showed that P2X7R expression on immune cells was responsible for the observed effect. Consistently, the inflammatory response is diminished only by a cell-type specific knockdown of P2X7 receptor on non-stationary immune cells. Since the results of BALF from patients with acute ARDS or pneumonia simulated the *in vivo* data after LPS exposure, the P2X7 receptor may be a new therapeutic target for treatment in acute respiratory distress syndrome (ARDS/ALI).

## INTRODUCTION

According to the revised Berlin definition, the term acute respiratory distress syndrome (ARDS/ALI) describes an acutely developing and progressive hypoxic respiratory failure characterized by bilateral lung infiltrates on chest radiograph that cannot be explained by heart failure or a hypervolemic state [3–5]. This definition includes the formerly separate concept of acute lung injury (ALI) within the ARDS entity. Since no specific therapy has been developed, the cornerstone of treatment still remains supportive care with ventilatory support. Though novel therapeutic strategies including extracorporal membrane oxygenation have led to improved survival, the mortality of the most severe ARDS cases remains unacceptably high around 50–60% [6, 7].

Among the various aetiologies of ARDS, infectious origins and sepsis are the most frequent predominantly triggering a hyperinflammatory subphenotype [8]. Besides being characterized by more severe inflammation, shock and metabolic acidosis, this hyperinflammatory phenotype is also associated with worse clinical outcome including a higher mortality despite usual therapeutic strategies [9].

In an experimental setting a bacterial hyperinflammatory phenotype of ARDS can be most effectively induced by lipopolysaccharide (LPS). LPS is an endotoxin originating from the cell wall of gram negative bacteria. The biological effects of LPS are triggered via the interaction with toll-like receptor 4 (TLR-4) [10] and the activation of the NLRP3 pathway eventually leading to the production of pro-inflammatory cytokines by various cell types.

In the past it has been demonstrated extensively that inflammation is associated with the release of nucleotides such as ATP, ADP, UTP or UDP into the extracellular space [11]. These nucleotides have the ability to mediate multiple cellular effects via signalling at P2-purinergic receptors. These receptors can be subdivided into metabotropic P2Y receptors (P2Y_1_, P2Y_2_, P2Y_4_, P2Y_6_, P2Y_11_, P2Y_12_, P2Y_13_ and P2Y_14_ isoforms) [12] and the P2X receptors which are ligand-gated ion-channels, (P2X_1_–P2X_7_) [13].

It has already been shown that the nucleotide ATP has an important role during the induction and maintenance of different inflammatory lung disorders [14]. Likewise, different cell types involved in bacterial infections of the lungs or ARDS development, e. g. neutrophils or macrophages express functional P2Y and P2X receptors. ATP for example promotes the release of proinflammatory cytokines, like IL-1β, from alveolar macrophages [15].

Due to its ubiquitous expression within the lung [16], and its importance in inflammatory responses, the P2X7 receptor is intensely investigated in lung injury. In addition to its important function in the pathogenesis of asthma, COPD and lung fibrosis, recently a role in ARDS development has been suggested [17].

This work focuses on the role of purinergic signalling in the pathogenesis of ALI/ARDS in animal models and patients. Our results indicate that a modification of P2X7 receptor signaling may be a new therapeutic target for treatment in acute lung injury (ARDS/ALI).

## RESULTS

### ARDS increases ATP-concentration in human BALF

**Figure 1:**
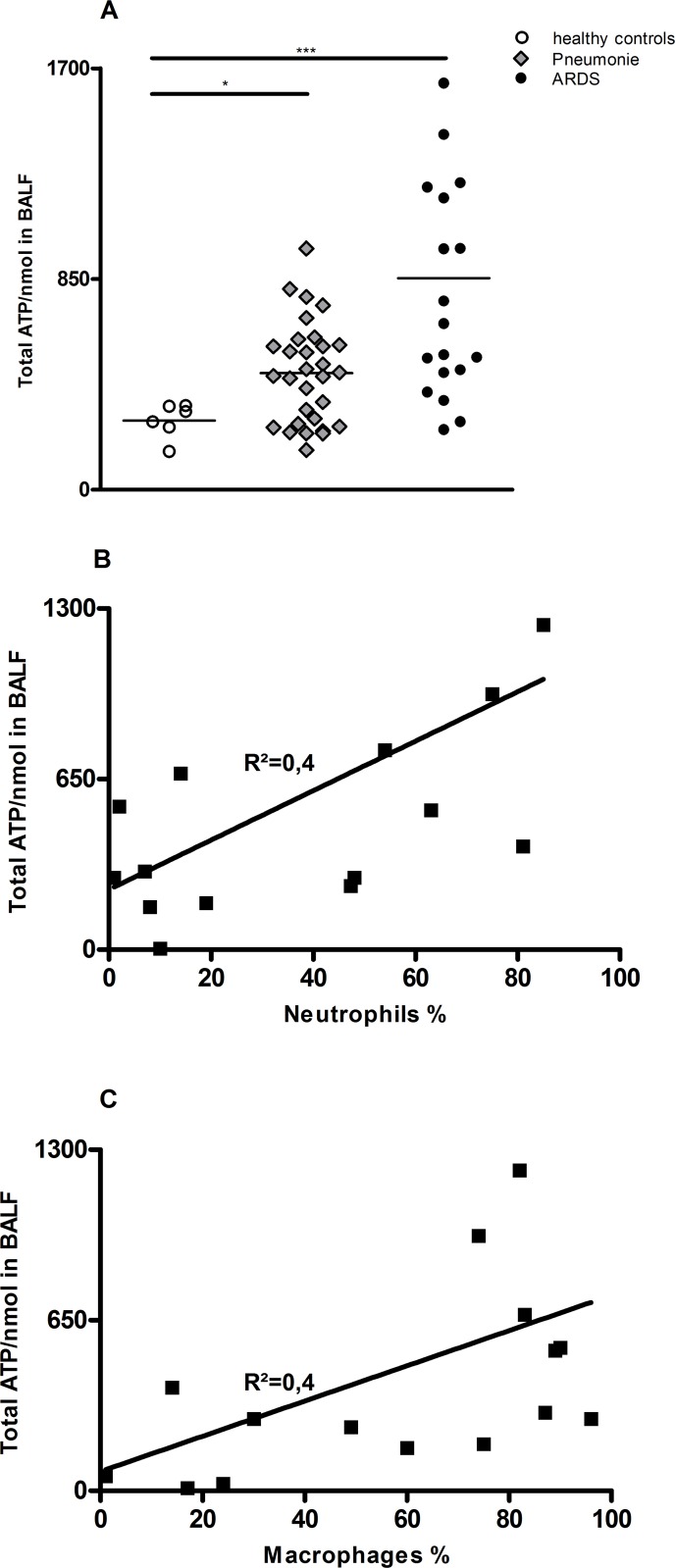
Increased ATP-Level in BALF of ARDS patients compared to healthy patients ATP concentrations in BALF were measured using ATPlite assay. (**A**) Significant increase in ATP levels in BALF of patients with pneumonia and ARDS compared to healthy controls. (**B** and **C**) Correlation between neutrophils (A) or macrophages (B) in BALF in % value and ATP concentration.

ATP concentrations were markedly elevated in BALF of patients with ARDS compared with normal control subjects (Figure 1A). In the total group of patients with ARDS, ATP concentrations in BALF correlated with the number of neutrophils and macrophages in BALF (r; P50). (Figure 1B and 1C). In attached tables the patient/BALF characteristics are listed (Supplementary Table 1).

### ATP accumulates in the lungs of mice with ALI/ARDS

To elucidate a possible role of P2R signalling in the observed ATP and neutrophil accumulation in patients with ALI/ARDS we analysed ATP-Levels and cell counts in BALF also in mice. Therefore C57BL/6 animals were exposed to LPS (300 µg/kg i.t.) or sterile PBS as a negative control. Animals were killed after different time points (24/48 hours) and the BALF was collected [14].

**Figure 2:**
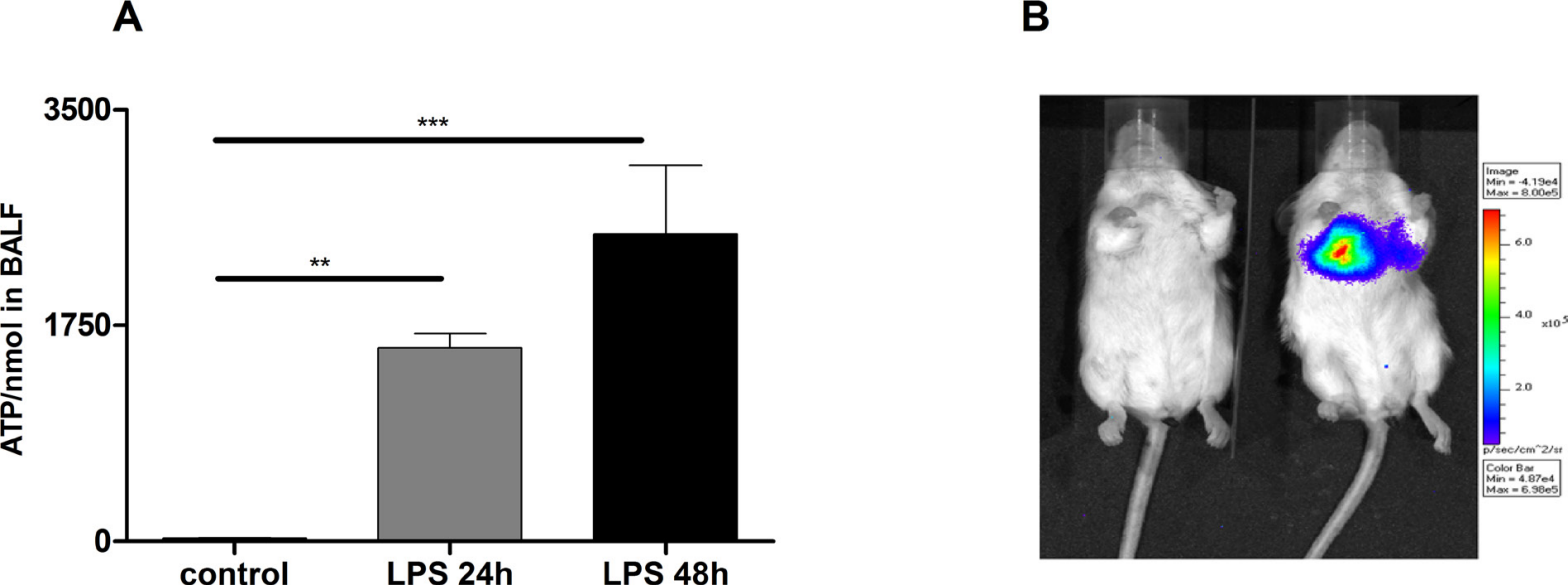
Increased ATP-Levels in the lungs of LPS treated mice (**A**) Significant increase of ATP in BALF after 24 h and 48 h of LPS exposure compared to controls. Animals were exposed to LPS (300 µg/kg/i.t.) and after 24 h and 48 h animals were killed and the BALF was collected. Data are means ± SEM, *n* = 5–10, ^*^p < 0.05; ^**^p < 0.01; ^***^p < 0.001. (**B**) Strongly enhanced *in vivo* extracellular ATP release in LPS exposed animals compared to controls. *In vivo* bioluminescence imaging using the IVIS 100 system.

LPS treated mice exhibited significantly increased ATP-levels compared to vehicle treated animals subjects (Figure 2A). Of note, ATP levels correlated with the total number of BALF cells and neutrophils.

To validate accumulation of ATP in the extracellular space in animals with experimental ALID/ARDS *in vivo*, extracellular ATP release was monitored by using bioluminescence imaging. As depicted in Figure 2B pulmonary ATP levels were strongly increased in mice exposed to LPS compared to negative controls.

### P2X7-receptor signaling contributes to LPS-induced ALI

**Figure 3:**
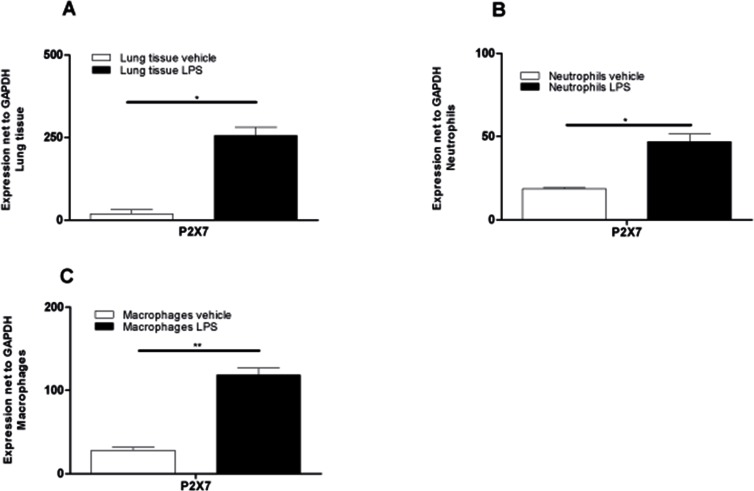
Significant upregulation of P2X7R-expression in lung tissue and BALF cells after LPS exposure Animals received LPS and vehicle on day 0 and were killed 24 hours later. Lung tissue and BAL-cells were collected and RNA was isolated. Relative expression of the P2X7R compared with GAPDH was analyzed using quantitative RT-PCR. (**A**) P2X7R subtype expression in lung tissue of PBS-exposed or LPS-exposed animals. (**B**) Expression of P2X7R on pooled BALF neutrophils (*n* = 10 LPS animals and *n* = 20 PBS animals). (**C**) P2X7R Expression on pooled BAL macrophages (*n =* 10 LPS animals and *n =* 20 PBS animals). Data are means ± SEM, *n* = 10–20, ^*^p < 0.05; ^**^p < 0.01; ^***^p < 0.

Purinergic signaling has been implicated in lung injury and in the pathogenesis of a wide range of respiratory disorders and diseases. Since it is known that especially P2X7R plays a crucial role in the pathophysiology of some lung inflammations, we analyzed the expression of P2X7R on lung tissue and BAL-cells by qt-PCR (in LPS-induced ALI-Model). Animals received either vehicle or LPS and were killed 24 hours after the treatment. Lung and BAL-cells were collected and mRNA was isolated. LPS exposure resulted in a significant up-regulation of P2X7R in total lung tissue, in BAL-macrophages and BAL-neutrophils compared to control mice (Figure 3A–3C). It is therefore important to note that LPS exposure–besides triggering elevated ATP-levels in BAL fluid–consistently acted on BAL-neutrophils by upregulating P2X7R expression and promoting their accumulation in BALF.

### P2X7R-Inhibition prevents the development of experimental ALI in animals

**Figure 4:**
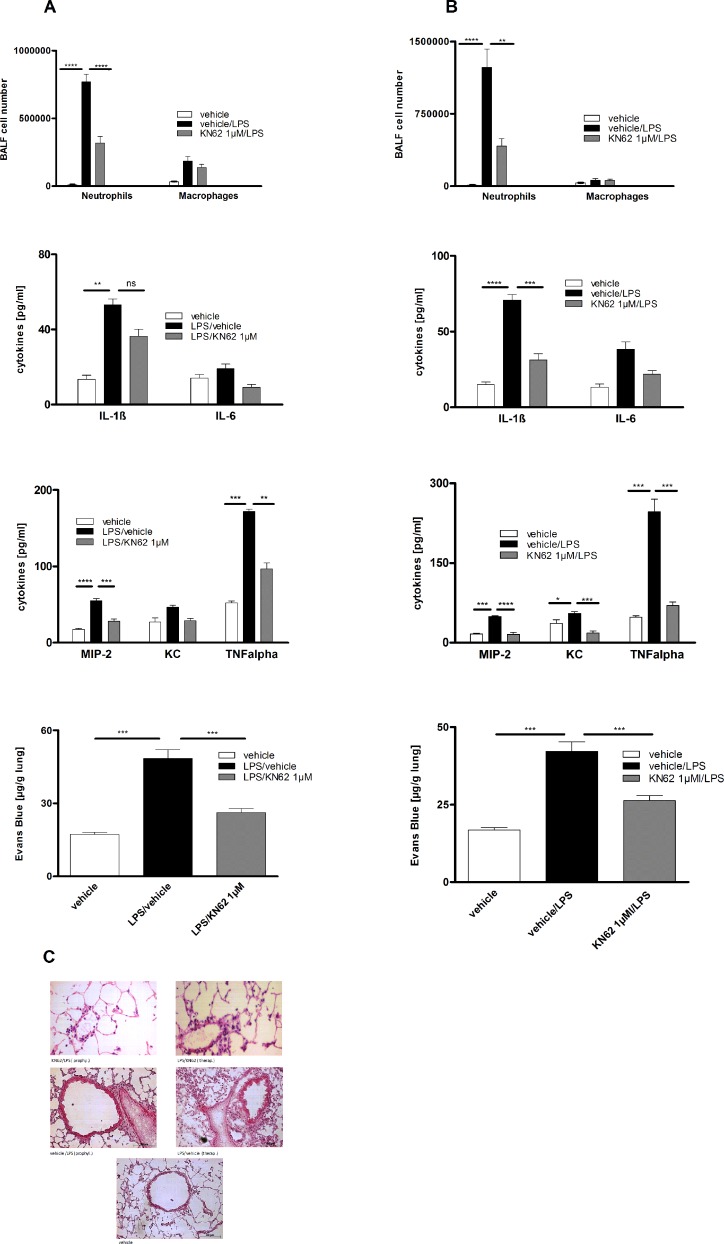

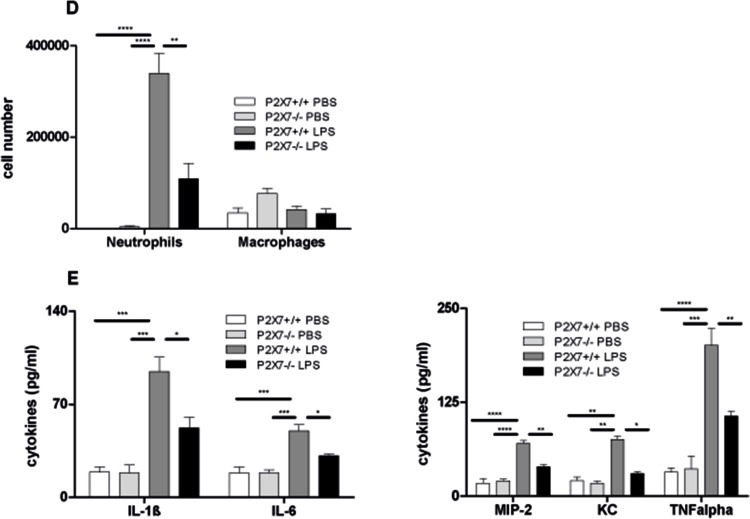
Effect of prophylactic or therapeutic administration of 1 µM KN62 and P2X7R-deficiency on LPS-induced ALI BALF cell differential count measured by flow cytometry. Concentration of IL-1ß, IL-6, KC, MIP-2 and TNF-α in BALF determined by ELISA. Plasma leakage determined spectrophotometrically 18 h after Evans blue dye albumin (20 mg/kg) was injected into the tail vein. (**A**) Therapeutic administration of 1 µM KN62 resulted in a non-significant reduction of BALF-cells, but in a significant decrease of cytokine levels (IL-1ß, MIP-2 and TNF-α) and a significant reduction in microvascular plasma leakage. (**B**) Prophylactic administration of 1 µM KN62 resulted in a significant reduction only of BALF-neutrophils and a decrease of cytokine levels (significant reduction for MIP-2 and TNF-α) as well as a significant reduction in microvascular plasma leakage. (**C**) Histology from lungs with therapeutically or prophylactically treated animals compared with untreated animal lungs showing reduced signs of ALI. (**D** and **E**) Wild-type and P2X7R−/− mice were left either untreated or exposed to LPS. Compared to wild-type P2X7R−/− mice exhibit a significant reduction of neutrophils in BALF (D) and all analyzed cytokine levels (IL-1ß, IL-6, MIP-2, KC, and TNF-α) (E). One representative experiment out of three is shown. Values are given as mean ± SEM. *n* = 5 mice in each group. ^*^p < 0.05, ^**^p < 0.01, ^***^p < 0.001 versus vehicle/LPS.

To assess the causative link between P2X7R upregulation and LPS-induced alterations, the selective P2X7R antagonist KN62, was administrated intratracheally in either a prophylactic or a therapeutic treatment regimen. In the prophylactic protocol animals received KN62 one hour before LPS exposure and were killed after 24 hours whereas in the therapeutic protocol KN62 was administered 1 h and 24 h after LPS exposure. Animals were killed 48 hours after LPS instillation in the therapeutic protocol. Both regimens led to a reduction of LPS-induced inflammation with respect to inflammatory cytokines (significant for IL-1β, MIP-2 and TNFα) and microvascular plasma leakage within the lung measured by spectrophotometry 18 h after Evans blue dye albumin injection. Accordingly, histologic features of LPS-induces lung inflammation were dampened. However, only the prophylactic administration of KN62 led to a significant reduction of neutrophils in BALF. The prevention of microvascular plasma leakage and lung inflammation was also more pronounced with this approach. Both regimens did not affect the macrophage cell count (Figure 4A–4C).

As proof of concept, we exposed P2X7R−/− or wild type animals either to LPS or left them untreated. 24 hours after LPS exposure, animals were killed and BALF was analyzed. As depicted in Figure 4D, 4E, P2X7R deficiency was associated with a decrease in LPS-induced lung inflammation mimicking the effects of KN62. The BALF-neutrophil count was significantly reduced whereas the macrophage cell count remained unaffected. Notably, the P2X7R knockout prompted a more profound change on the selected cytokines levels. A significant reduction was achieved not only for IL-1β, MIP-2 and TNFα, but also for IL-6 and KC.

### Cell-type specific P2X7R-knockout in hematopoietic cells results in reduced lung inflammation after LPS exposure

**Figure 5:**
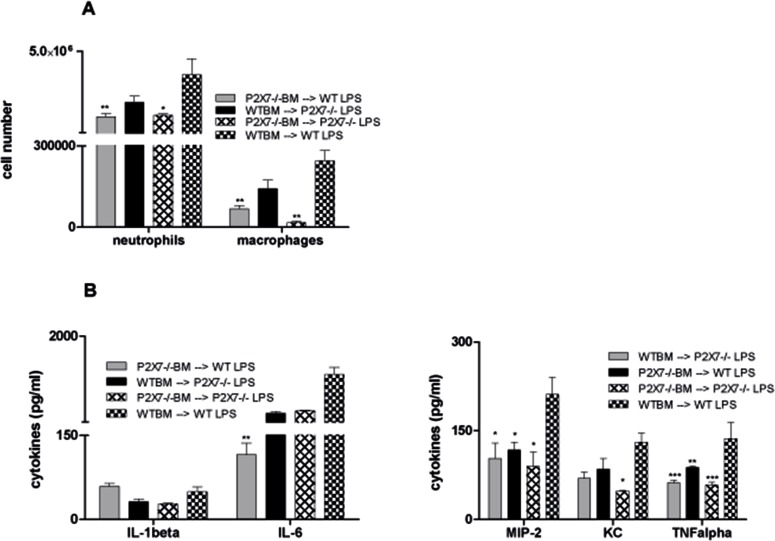
Role of P2X7R-expression on the hematopoietic system in LPS-induced lung inflammation The different BM chimera animals were exposed to LPS. 24 hours later animals were killed and BALF was analyzed by flow cytometry. Cytokine levels were determined by ELISA. (**A**) Significant decrease of neutrophils and macrophages in BALF of P2X7R−/− animals and WT mice with a lack of P2X7R expression in the hematopoietic system compared to WT mice. (**B**) No effect on IL-1β-levels. Significant reduction of IL-6 levels only in WT mice with a lack of P2X7R expression in the hematopoietic system and of KC only in P2X7R−/− animals. Significant MIP-2 and TNF-α reduction in all P2X7R−/− chimera with largest effects in WT mice with a lack of P2X7R expression in the hematopoietic system and complete P2X7R−/− animals. ^*^p < 0.05, ^**^p < 0.01, ^***^p < 0.001 versus vehicle/LPS.

Consecutively, the individual contribution of structural versus hematopoietic cells in the P2X7R-dependent pathogenesis of LPS-induced lung injury was investigated by bone marrow transplantation experiments. Different BM chimera animals (WT → WT, P2X7R−/− → WT [hematopoietic system P2X7R−/−], WT → P2X7R−/− [non-hematopoietic system P2X7R−/−] and P2X7R−/− → P2X7R−/−) were either left untreated or exposed to LPS and then analyzed for lung inflammation. Compared to WT chimera and non-hematopoietic P2X7R −/− mice, animals with a lack of P2X7R expression in the hematopoietic system displayed a significant decrease of BALF cells after LPS exposure. This effect was observed for neutrophils as well as for macrophages (Figure 5A). The analyzed cytokine levels were diversely affected in the individual chimera experiments, although compared to WT a decrease was seen for all cytokines except for IL-1β. IL-6 levels were significantly reduced only in mice with a lack of P2X7R expression in the hematopoietic system, KC levels only in complete P2X7R −/− mice. For MIP-2 and TNFα a significant change was achieved in all P2XR7 −/− chimera with the largest drop in hematopoietic P2X7R−/− and complete P2X7R−/− chimera (Figure 5B). This clearly demonstrates the crucial role of hematopoietic P2X7R-signalling for LPS-induced lung injury.

**Figure 6:**
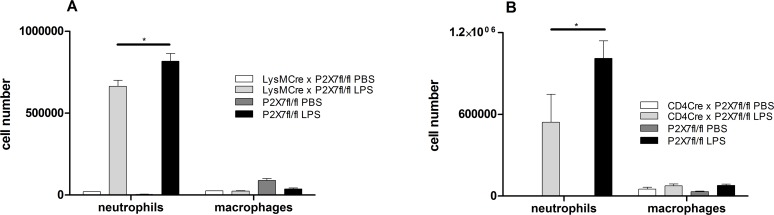
BALF cell counts in conditional CD4-Cre × P2X7fl/fl and LysM-Cre × P2X7fl/fl animals The animals were exposed with LPS or PBS and 24 hour later animals were killed. BALF was analyzed by flow cytometry. ^*^p < 0.05. (**A**) Significant reduction of the neutrophil count in P2X7Rfl/fl crossed with CD4-Cre and LysM-Cre animals. The number of macrophage was not significantly affected.

To further characterize the cell-specific influence of P2X7R-signalling in the pathogenesis of ALI/ARDS, we repeated the experiments with conditional P2X7Rfl/fl mice crossed with CD4-Cre animals (lymphocytes), LysM-Cre animals (macrophages/neutrophils) and CCT-Cre animals (airway epithelial cells). All conditional mice were treated intratracheally with LPS. 24 hours later mice were killed and the BALF was collected. While CCT-Cre x P2X7Rfl/fl animals did not exhibit any protective properties in ALI (data not shown), a significant reduction of the neutrophil count in BALF was achieved in P2X7Rfl/fl crossed with CD4-Cre and LysM-Cre animals. The number of macrophage was not significantly affected (Figure 6A, 6B). This clearly points to a pivotal role of P2X7R-signalling through non-stationary lymphocytes and neutrophils in the development of ALI after LPS exposure.

### PMN migration into the interstitium and BALF

**Figure 7:**
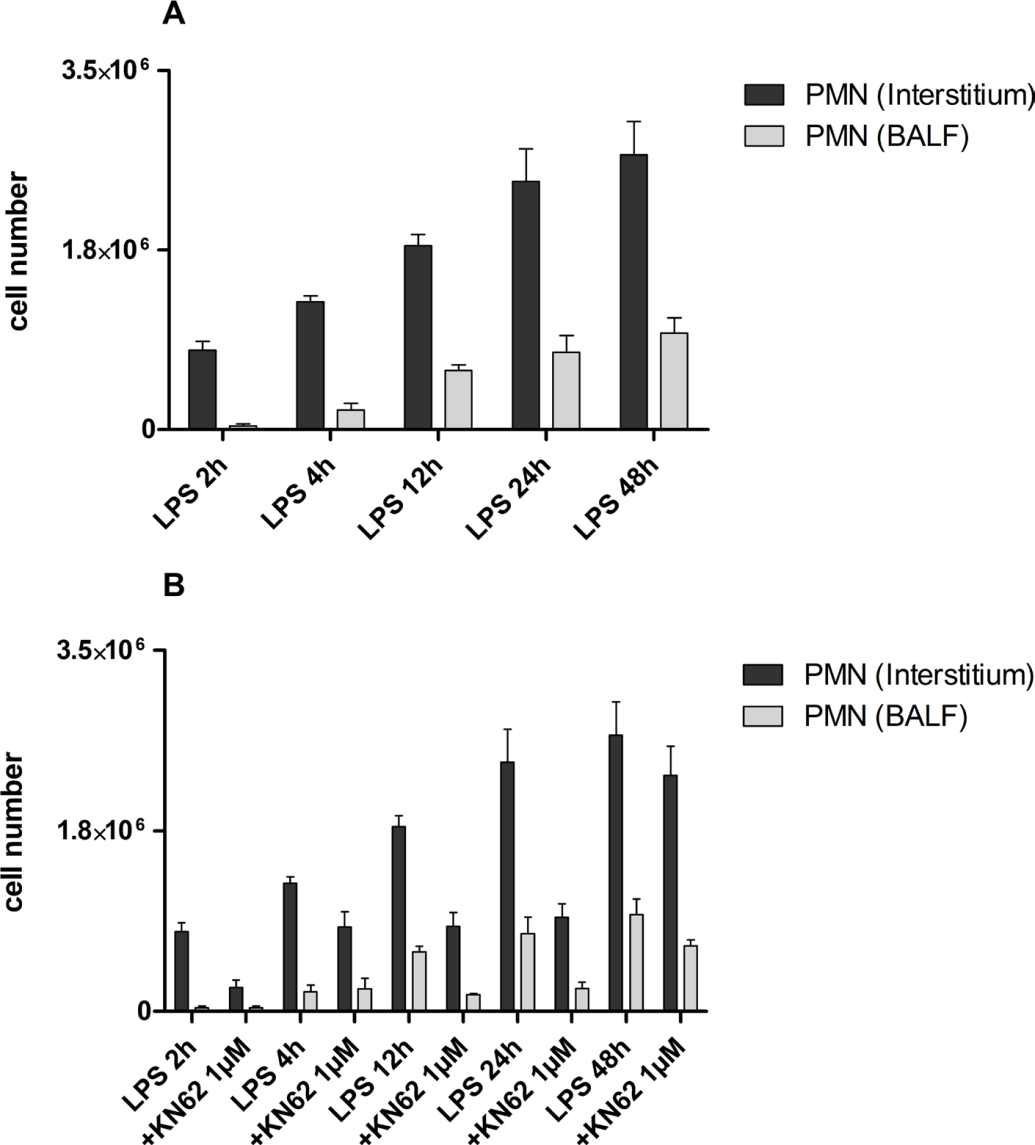
PMN migration into the interstitium and BALF Mice with LPS exposure and treatment with KN62 (1 µM) were injected intravenously with Alexa 633-labeled rat anti-mouse GR-1 antibody and allowed to circulate for 5 min to bind intravascular PMN. After 5 min, mice were killed and BALF and lungs were harvested. BALF was determined by flow cytometry and the lungs were digested. These recovered cells were counted and determined by flow cytometry. ^*^p < 0.05, ^**^p < 0.01, ^***^p < 0.001 versus vehicle/LPS. (**A**) Continuously increasing number of PMNs in the interstitium and in the BALF (alveolar space) after LPS-inhalation during the 48 h observation period. (**B**) Decreased PMN accumulation in KN62-treated mice compared to control after LPS-inhalation. There was no further increase of PMN accumulation in KN62-treated mice after 4 until 24 hours.

To further characterize the role of P2X7R signaling on non-stationary immune cells in a spatio-temporal manner during ALI, quantitative polymorphonuclear neutrophil (PMN) migration in the different compartments of the lung (interstitial, alveolar space) was evaluated *in vivo* over 48 h in control mice and KN62-treated mice. As depicted in Figure 7A for control mice, the number of PMNs continuously increased in the interstitium and in the BALF (alveolar space) after LPS-inhalation during the 48 h observation period. While sharing the same dynamic, PMN accumulation in the interstitium exceeded that of the alveolar space.

In KN62-treated animals PMN-accumulation at all time points was significantly lower than in control mice. Although the initial migration of PMN to interstitium and alveolar space during the first four hours after LPS-inhalation was not completely abolished, accumulation of PMNs peaked at four hours and did not further increase until 24 hours (Figure 7B).

## DISCUSSION

In recent years, the P2X7-receptor and its endogenous ligand ATP have gained attention as initiators of inflammation associated with several chronic diseases, such as COPD, IPF and ARDS/ALI [18–20]. This receptor subtype is ubiquitously expressed in almost all tissues and organs of the body with the highest distribution in the immune cells of monocyte-macrophage origin. Classically, P2X7 receptor is involved in apoptotic cell death, and it is well known that extracellular ATP ligation to this purinergic receptor serves as an important secondary stimulus, which is also considered as danger signal for the interleukin (IL)-1β cleavage and secretion from pro-inflammatory cells.

ALI/ARDS is pathophysiologically characterized by an acute alveolar damage, an increased permeability of the microvascular-barrier, leading to protein-rich pulmonary edema and subsequent impairment of arterial oxygenation and respiratory failure [1, 21–24]. LPS exposure induces a bacterial hyperinflammatory phenotype of ARDS and results in a high recruitment of neutrophils to the lung as seen by increased BALF-neutrophilia and PMN-Trafficking in lung compartiments [25].

This article demonstrated a functional role for the purinergic receptor P2X7 in LPS-induced Inflammation in the lung *in vivo*. We verified that the ATP-Levels in BALF of actual ARDS patients are massively elevated compared to control patients. Furthermore, ATP concentrations were positively correlated with BALF neutrophil counts. Of note, as previously reported for COPD patients, ATP levels correlated with the total number of BALF cells and neutrophils. This result suggested a functional connection between extracellular ATP and neutrophils in the airways [26].

The results of the ARDS patients could be well mimicked in the mouse-model. LPS-exposure revealed an accumulation of ATP not only in the BALF but also in the lungs of animals *in vivo* (bioluminescence method). Additionally P2X7 was upregulated in macrophages, neutrophils and lung tissue. Intrapulmonary application of KN62 (specific P2X7 antagonist) reduced the amount of neutrophils and the production of pro-inflammatory cytokines. The experiments with P2X7−/− mice, chimera animals and celltype-specific P2X7−/− mice proved that the P2X7R on immune cells rather than lung tissue cells may play a central role in LPS-induced lung inflammation.

It is well known that P2X7R is involved in the recruitment of neutrophils to the lungs and it has recently been shown that P2X7R dependent modulation of αMβ2-integrins (MAC-1) expression in neutrophils plays an important role for this effect [27].

In this study we demonstrated that selective inhibition of the P2X7R with KN62 as well as P2X7R deficiency was associated with reduced pulmonary inflammation. Intrapulmonary application of KN62, before or after LPS administration, reduced the amount of neutrophils, the production of pro-inflammatory cytokines and decreased the vascular leakage in the lung. As vascular leakage is a very important feature for pulmonary edema in ALI/ARDS [28], the significant improvement after P2X7 antagonist application supports the crucial role of ATP signaling in ALI/ARDS development.

Additional to the modulation of MAC-1 expression in neutrophils, P2X7R inhibition can also indirectly affect neutrophil migration via a suppression of neutrophil-attracting cytokines [29]. Indeed the concentrations of neutrophil-attracting cytokines, KC and MIP-2, were strongly reduced in LPS-induced ARDS mice treated with KN62 (P2X7R antagonist), which could also explain a decrease in BAL neutrophilia in these animals as a result of diminished PMN migration in to the lung.

Alveolar deposition of LPS normally causes a significant influx of neutrophils followed by macrophages into lung interstitium and alveolar space [30]. Consistent with previous work, the P2X7R knockout mice in our study were protected from LPS-induced ALI. Already a few years ago the connection between P2X7 activation and IL-1ß production has been elucidated [31]. The reduced migration of neutrophils into the interstitium could also be the result of a reduced IL-17 expression due to inhibition of P2X7-signalling since IL-17 is known to mediate the recruiting of neutrophils by increasing chemokine production [32, 33]. Even though we did not look for IL-17 levels in our study, it is known that IL-17 is upregulated among others by IL-1ß and Il-6 [34]. Indeed, Wang and colleagues confirmed a P2X7-dependent IL-17 upregulation in LPS-induced ALI.

As P2X7R expression was upregulated both in lung tissue and in immune cells, we intended to clarify whether structural cells or immune cells primarily contribute to this results. Therefore, we performed experiments with P2X7R chimera mice. Only the reconstitution of WT animals with P2X7−/− bone marrow resulted in high inhibition of LPS-induced inflammation. This indicated a pivotal role of P2X7R signaling in hematopoietic cells for the development of ARDS/ALI. For this reason we used cell-type-specific P2X7R-KO mice to confirm the chimera results. We specified that non-stationary lymphocytes and neutrophils are mainly involved in LPS-induced inflammation.

Consistent with our results a recent study has shown that the LPS-induced lung injury leads not only to an upregulation of P2X7 but also of the downstream targets NLRP3, ASC and the active form of caspase 1. The same group demonstrated that selective inhibition of P2X7 after LPS-induced lung injury caused a downregulation of NLRP3, ASC and the active form of caspase 1 whereas its precursor form (pro-caspase 1) was not affected [5].

An indirect method to assess the question of polymorphonuclear leukocyte accumulation in the lung tissue represents the measurement of MPO activity. This was done in a recent study by Lv *et al.* who reported increased MPO activity in the lung tissue after LPS challenge. This effect could be hampered by application of Tenuigenin (TNG), a traditional chinese medicine with anti-oxidant and anti-inflammatory properties [35]. In contrast to P2X7R antagonists, TNG seems to work upstream of the NLRP3 inflammasome by attenuating the activation of the MAP-kinases ERK, p38 and by inhibiting NF-kB activation.

In conclusion, consistent with recent results of other groups our results confirm the pivotal role of the P2X7 pathway in the inflammatory response that leads to ALI/ARDS. Different from approaches with cytotoxic agents that interfere with the synthesis/expression of the NLRP3-inflammasome complex, the inhibition of the P2X7 receptor bears the advantage of specifically blocking the production of proinflammatory cytokines without affecting anti-inflammatory signals and cell replication.

## MATERIALS AND METHODS

### Animal studies

#### Mice

C57/Bl6 mice (6–8 weeks old), CCT-Cre × P2X7fl/fl, LysM-Cre × P2X7fl/fl, CD4-Cre × P2X7fl/fl mice were bred at the animal facilities of the Medical Center–University of Freiburg under specific pathogen free conditions. All experiments were performed according to the institutional guidelines of the animal ethics committee of the German government.

### Model of LPS-induced acute lung inflammation (ALI/ARDS)

For induction of acute lung inflammation, mice (C57/Bl6) were anesthetized by intraperitoneal injection of Ketamin-Rompun (4 mg/kg and 80 mg/kg) and received an intratracheal (i.t.) injection of LPS (Escherichia coli Serotype 026:B6, Sigma, Steinheim, Germany) in a total volume of 50 µl in sterile PBS to a final dose of 300 µg/kg. The control mice group received an intratracheal (i.t.) injection of PBS. The dose was based on previous studies [36, 37].

### Treatment with purinergic receptor antagonists

The indicated concentration of KN62 (1 µM) or vehicle diluted in 80 µl PBS was administered i.t. 1 hour before (prophylactic protocol) or 24 hours after (therapeutic protocol) the LPS instillation.

### Collection of lung tissues, bronchoalveolar lavage fluid and BALF-cells

24 hours after the last exposure of LPS and purinergic receptor antagonist, animals were sacrificed by intraperitoneal (i.p.) injection of Thiopental (200 mg/kg) and exsanguinated. BAL was performed with 3 × 1 ml sterile PBS supplemented with 0.1 mM sodium EDTA, followed by lung resection for lung histology and qPCR analysis. Bronchoalveolar lavage fluid (BALF) was kept on ice until further processing. For histology, lungs were stored in OCT freezing medium and for qPCR, lung pieces were homogenized and stored in Qiazol (Qiagen).

BALF neutrophils and macrophages were isolated by use of FACS sorting. Therefor BAL cells were stained for 20 minutes with anti-Ly-6G (Gr-1) FITC and anti-mouse F4/80 PE, in PBS containing 0.5% BSA and 0.01% sodium azide and subsequent isolated with FACS sorter.

### ATP measurements in BALF of mice

The ATP levels were measured in BALF of mice immediately after collection using ATPlite assay (Perkin Elmer) according to the instructions of the manufacturer. The cell lysis step was omitted to avoid any contamination of intracellular ATP as previously described [38].

### *In vivo* detection of extracellular ATP release

Mice (C57/Bl6) received an i.t. application of 1 × 10^6^ cells with membrane-targeted luciferase (PME cells). Additionally, animals received intratracheal LPS or PBS as described above.

On the next day animals were anesthetized followed by an i.t. injection of luciferin. Subsequently *in vivo* bioluminescence imaging using the IVIS 100 system was performed, as previously described [39].

### Total cell count and flow cytometry

BALF was centrifuged (5 min, 1500 rpm), and supernatants were stored at −20° C for subsequent analysis of cytokine levels. Cell pellets were resuspended in 200 µl PBS for total cell counting using a hemacytometer. The differential cell count analysis were carried out by flow cytometry (FacsCalibur BD Bioscience; San Diego, CA, USA) as previously described [18] Briefly, mouse BALF cells were incubated with unlabeled anti-CD16/CD32 to block Fc receptors and stained for 20 minutes with anti-CD11c APC, anti-Ly-6G (Gr-1) FITC, anti-CD3e PE-Cy7, anti-CD45R (B220) PE-Cy7 and anti-mouse F4/80 PE, in PBS containing 0.5% BSA and 0.01% sodium azide. Differential cell counts were analyzed using the software Cellquest version 3.3 (BD Bioscience, San Diego, CA, USA) and FlowJo version 10 (TreeStar Inc., Ashland, OR, USA) [39].

### Analysis of cytokine levels

Concentrations of Interleukin-1beta (IL-1β), Interleukin-6 (IL-6), macrophage inflammatory protein-2 (MIP-2), keratinocyte-derived chemokine (KC) and tumor necrosis factor alpha (TNF-α) were measured in BALF using enzyme-linked immunosorbent assays (ELISA) (R&D Systems, Duoset, Minneapolis, Minn, USA), according to the manufacturer’s instructions. The detection limit was 2 pg/ml. Samples with values below the detection limit were assigned 1 pg/ml as cytokine concentration.

### Histology

Frozen lungs were cut and stained with haematoxylin and eosin. The density of neutrophils in the lung parenchyma was obtained as previously described [40–43]. Briefly, 20 photomicrographs of lung parenchyma (excluding areas of pulmonary vessels) were randomly obtained at 400× magnification. The area of the whole photograph and the area of light area (air area) were calculated using the Software Image Pro Plus 4.0. (Media Cybernetics, Inc., Rockville, MD, USA) Subtracting the air area from the total photograph area we obtained the parenchymal tissue area. Then, according to the morphological criteria, the number of neutrophils was counted in the tissue area. The results were depicted as number of neutrophils per square millimeter of parenchymal tissue (neutrophils/mm^2^).

### Quantitative PCR analysis of purinergic receptors

Total RNA was extracted from the cells using Qiazol (Qiagen). cDNA synthesis was performed with random primers and First Strand cDNA Synthesis-Kit (Thermo-Fisher Scientific GmbH, Schwerte, Germany). Primers for the different murine purinergic receptors were used to detect the expression of the mRNAs. Quantitative PCR was performed with Taqman Universal PCR Mastermix (Applied Biosystems, Foster City, USA) and pre-formulated primers and probe mixes (Assay on Demand, Applied Biosystems). PCR conditions were 2 min at 50° C, 10 min at 95° C, followed by 45 cycles of 15 s at 95° C and 60° C for 1 min using a thermal cycler (iCycler, Biorad, Hercules, USA). Glyceraldehyde-3-phosphate dehydrogenase (*Gapdh*) was used as reference gene. Primerdesign and relative quantifications were done as previously described [44]; primer sequences are available upon request.

### Plasma leakage assay

Plasma vascular leakage was examined as previously described [45]. Briefly, Evans blue dye conjugated to albumin (20 mg/kg) was injected into the tail vein of mice. 30 minutes later the mice were sacrificed and the lungs were perfused with PBS supplemented with 5 mM EDTA. Perfused lungs were excised en bloc, dried, weighed and snap frozen in liquid nitrogen. The whole lung was homogenized in PBS (1 mL/100 µg tissue) prior to incubation in formamide at 60° C for 18 hours. The optical density of the supernatant was determined spectrophotometrically at 620 nm after centrifugation at 5,000 × g for 30 minutes. The concentration of the extravasated Evans blue in lung homogenate was calculated against the standard curve and the results expressed as µg of Evans blue dye per gram lung tissue [45].

### Polymorphonuclear neutrophil (PMN) trafficking in the Lung

At different timepoints after LPS exposure and treatment with KN62 mice were injected intravenously with Alexa 633-labeled rat anti-mouse GR-1 (10 µg) antibody and allowed to circulate for 5 min to bind intravascular PMN. After 5 min, mice were killed. After performing BAL, lungs were harvested in total. Lungs were minced and digested with 125 U/ml collagenase type XI, 60 U/ml hyaluronidase type I-s, and 60 U/ml DNase (all Sigma) at 37° C for 30 minutes. Cell suspension was obtained by passing digested lungs through a cell strainer and the suspension was centrifuged for 10 min at 300 × g. The pellet was lysed with lysis buffer to remove erythrocytes and centrifuged again. Pellet was resuspended in buffer and cells were counted with a haemocytometer [25].

### P2X7-Signalling on haematopoietic system vs. structural cells

Wildtype (WT) or P2X7R−/− recipients were given 5 × 10^6^ WT or P2X7R−/− bone marrow (BM) cells intravenously after lethal irradiation with 900 cGy (2 × 450 cGy). The following donor-recipient pairs were combined: WT→WT, P2X7R−/−→WT (hematopoietic system: P2X7R−/−), WT→P2X7R−/− (non-hematopoietic system: P2X7R−/−), P2X7R−/−→P2X7R−/−. Six to eight weeks later the animals were challenged with LPS and the classical features of ARDS were determined as described above [46–48]. In addition, we used conditional P2X7−/− mice (crossed with CD4-Cre (lymphocytes), LysM-Cre (macrophages/neutrophils), CCT-Cre (airway epithelial cells) animals) to see which type of cells play a pivotal role in the pathogenesis of ALI/ARDS. Animals received an i.t. injection of LPS (300 µg/kg) and were killed 24 hours later.

### *In vitro* studies

#### BALF of patients with ARDS

BAL fluids derived from 12 patients with ARDS, 30 patients with pneumonia and 6 healthy control subjects were collected at the Medical Centre - University of Freiburg. Patients with malignancies or were excluded. In the control group, no cardiac or pulmonary medications were allowed. The study was approved by the local ethics committee of Freiburg. All participants gave their written informed consent. Bronchoscopy and BAL as well as sample preparation are strictly standardized at our institution. BAL was performed as reported previously 1. After analysing CAT scans BAL was mainly carried out in the middle lobe or the lingula unless CAT-scans showed absence of pathologic changes in these areas of the lung. 300 ml of prewarmed sterile saline (0.9% NaCL) were instilled in 20 ml aliquots. After each instillation, the fluid was gently suctioned. The BAL aliquots were pooled and collected in a siliconized glass bottle which was kept on ice and immediately transported to the laboratory. BAL with more than 1% of squamous epithelial or ciliated cells or a recovery rate of less than 30% were not accepted. The BAL fluid was filtered through two layers of cotton gauze. The cells were centrifuged at 500 × g and then washed 3 times with phosphate buffered saline at + 4° C. Cell count and cell viability were assessed after staining with tryptan blue, using a Bürker Chamber (Marienfeld, Germany). Cell differentials were determined by counting a minimum of 200 cells on a cytocentrifuge preparation using a Shandon Cytospin II (Pittsburg, PA) stained by HEMACOLORTM (e. Merck, Darmstadt, FRG). Air dried smears were prepared as described 2. BAL volume was categorized in small (SV: 100 to 200 ml; median: 120 ml; *n =* 35), large (LV: >200 ml; median: 300 ml; *n =* 203; LV). All other technical conditions were kept constant [49, 50].

### Statistical analysis

Data are expressed as mean ± SEM and compared using Bonferroni comparison test and followed by one-way ANOVA (GraphPad Prism 5 Software, San Diego, CA, USA.)

## SUPPLEMENTARY MATERIALS TABLE



## Abbreviations

ALI: Acute lung injury
ARDS: Acute respiratory distress syndrome
ATP: Adenosine triphosphate
ADP: Adenosine diphosphate
BALF: Broncho-alveolar-lavage-fluid
COPD: Chronic obstructive pulmonary disease
EDTA: Ethylendiamintetraacetate
FACS: Fluorescence-activated cell sorting
H&E: Hematoxylin and Eosin
IL: Interleukin
i.p.: Intraperitoneal
i.t.: Intratracheally
KN62: (S)-5-Isoquinolinesulfonic acid 4-[2-[(5 isoquinolinylsulfonyl) methylamino]-3-oxo-3-(4-phenyl-1-piperazinyl)propyl] phenylester, 1-[N,O-bis(5-Isoquinolinesulfonyl)-N-methyl-L tyrosyl]-4-phenylpiperazine; PME cells membrane-targeted luciferase cells
P2R: Purinergic-type 2-receptor
SPF: Specific Pathogen Free
TLR-4: Toll-like receptor 4
UDP: Uridine diphosphate
UTP: Uridine triphosphate
WT: Wildtype
