# Supplementary material for: Extracellular ATP is a danger signal activating P2X7 receptor in a LPS mediated inflammation (ARDS/ALI)
Source: Oncotarget. 2018 Jul 17;9(55):30635–48. doi: 10.18632/oncotarget.25761 (PMC6078145; doi:10.18632/oncotarget.25761)
Supplement: Supplementary file 1 [file oncotarget-09-30635-s001.pdf]

Extracellular ATP is a danger signal activating P2X7 receptor in a LPS mediated inflammation (ARDS/ALI)

SUPPLEMENTARY MATERIALS

Supplementary Table 1: Patient and BALF characteristics (healthy, Pneumonie, ARDS): Cicko *et al.* 2018

	Healthy controls	Pneumonie	ARDS
**Patient characteristics**	6	30	19
No. of subjects	2/4	18/12	17/3
Sex, M/F	43,8 (15,2)	61,7 (16,8)	60,5 (17,0)
Age, jr (SD)	0 (0)	4,2 (9,7)	47,0 (29,0)
Pack-jears (SD)			
**BALF characteristics**			
10^6 cells/100 ml BALF (SD)	5,1 (2,9)	14,9 (9,5)	22,8 (21,4)
Macrophages, % (SD)	99,6 (0,5)	64,3 (26,0)	48,3 (33,3)
Neutrophils, % (SD)	0,3 (0,5)	7,7 (12,1)	39,1 (31,9)
Eosinophils, % (SD)	0 (0)	4,0 (8,9)	1,8 (2,5)
